# Rescue Cerclage by McDonald’s Technique at 18 Weeks for Cervical Insufficiency With Intravaginal Amniotic Sac: A Case Report

**DOI:** 10.7759/cureus.53264

**Published:** 2024-01-30

**Authors:** Pankaj Salvi, Vidya Gaikwad, Ashton Dsouza, Sravani Ankem

**Affiliations:** 1 Obstetrics and Gynaecology, Dr. D. Y. Patil Medical College, Hospital & Research Centre, Pune, IND

**Keywords:** recurrent pregnancy loss, intravaginal bulging, chorioamnionitis, preterm premature rupture of membranes (pre-prom), cervical cerclage

## Abstract

Recurrent pregnancy loss, premature birth, and associated complications exhibit a multifactorial etiology and persist as substantial challenges during pregnancy, despite the notable advancements in the medical field. Among several factors, cervical insufficiency or incompetence emerges as a prominent causal factor, characterized by painless softening and shortening of the cervix associated with absent contractions. The implementation of emergency cerclage represents a pivotal intervention in mitigating preterm birth among individuals with advanced cervical insufficiency. By extending gestational age, this procedure increases the likelihood of neonatal survival without elevating the risk of chorioamnionitis or preterm rupture of the membranes. In this study, an antenatal woman presented with advanced changes in the cervix along with intravaginal bulging amniotic membranes at 18 weeks and underwent a rescue cervical cerclage, resulting in a successful pregnancy.

## Introduction

The primary function of the cervix is to uphold the pregnancy until fetal maturation through mechanical strength and as a barrier to ascending infection [1]. While the etiology of preterm delivery is undoubtedly multifaceted, there is growing literature on the significance of cervical dysfunction in this process [2]. Emergency cerclage procedures are primarily performed in cases involving women who present during the mid-trimester with symptoms indicative of threatened abortion or preterm delivery. These symptoms typically manifest as abdominal pain or vaginal discharge [3]. Upon examination, the fetal membranes are frequently observed, protruding through the external os in a characteristic *hourglass* configuration. The implementation of emergency or *rescue* cerclage is considered after the exclusion of other potential causes of second-trimester miscarriage [4]. The patient is positioned in the lithotomy position, and the protruding forewaters are delicately reduced, followed by the application of a suture to close the cervix. Rescue cervical cerclage has exhibited advantages in prolonging pregnancy to a stage of viability in women who present with cervical dilation and the protrusion of fetal membranes in the second trimester. When performed under emergency conditions with strict aseptic measures, this intervention holds the potential to significantly extend pregnancy and enhance the chances of achieving a viable pregnancy outcome [5,6]. However, it is imperative to communicate to the patient the possible risk of infection associated with the fetal membrane being exposed to vaginal bacteria during the procedure.

## Case presentation

A primigravida, 23 years of age, with a gestational age of 18 weeks and four days as determined by the first-trimester ultrasound, presented to the labor room with ultrasound suggestive of incompetent os with a cervical length of 0.72 cm and open os. Notably, the patient did not report any history of abdominal pain, vaginal bleeding, or abnormal vaginal discharge. Her menstrual cycles were irregular, occurring every two to three months with bleeding lasting for one to two days. The current pregnancy was achieved through a three-month course of ovulation induction. The patient had a medical history of polycystic ovary syndrome and had been intermittently on treatment with oral contraceptive pills. There were no other significant past medical issues, and her personal and family medical history did not reveal any noteworthy findings. Vital signs were within normal limits, and the fundal height corresponded with the expected period of gestation.

A per-speculum examination was performed, revealing the presence of membranes protruding through the external os into the vaginal cavity. The external os was found to be 2 cm dilated, and cervical effacement ranged between 20% and 30%. Subsequently, the patient was admitted, and routine investigations were initiated. The treatment plan included administering injection hydroxyprogesterone caproate and initiating tablet natural progesterone at 200 mg SR OD, accompanied by injectable antibiotics such as ceftriaxone 1 g IV and injection metronidazole 500 mg IV.

**Figure 1:**
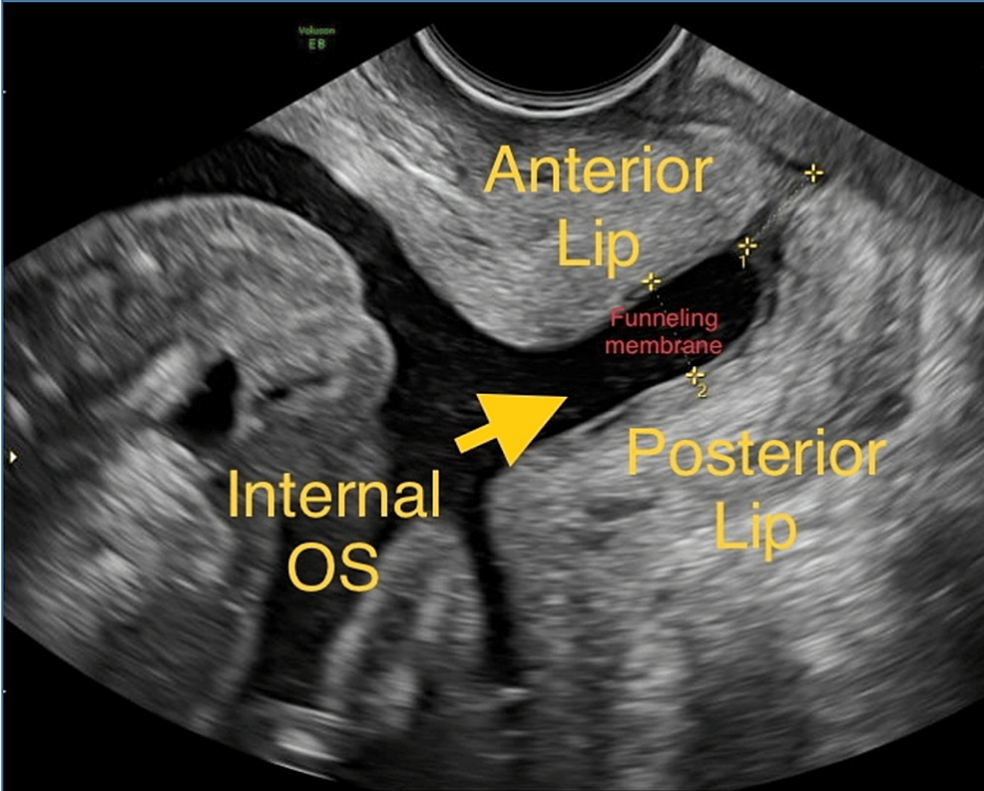
Ultrasound image showing short cervix with funneling of membranes.

Upon ultrasound examination, a single live fetus in breech presentation corresponding to 17 weeks and six days was identified. The placenta was situated anteriorly at the fundus, and the estimated effective fetal weight was measured at 213 g. An anomaly scan was performed, revealing normal findings. However, the cervical length was measured at 7.2 mm, indicating funneling of membranes and an open internal os (Figure 1). These observations collectively suggested cervical incompetence and raised concerns about a threatened abortion. The patient’s condition required careful monitoring and appropriate management to address the risk of pregnancy loss.

**Figure 2:**
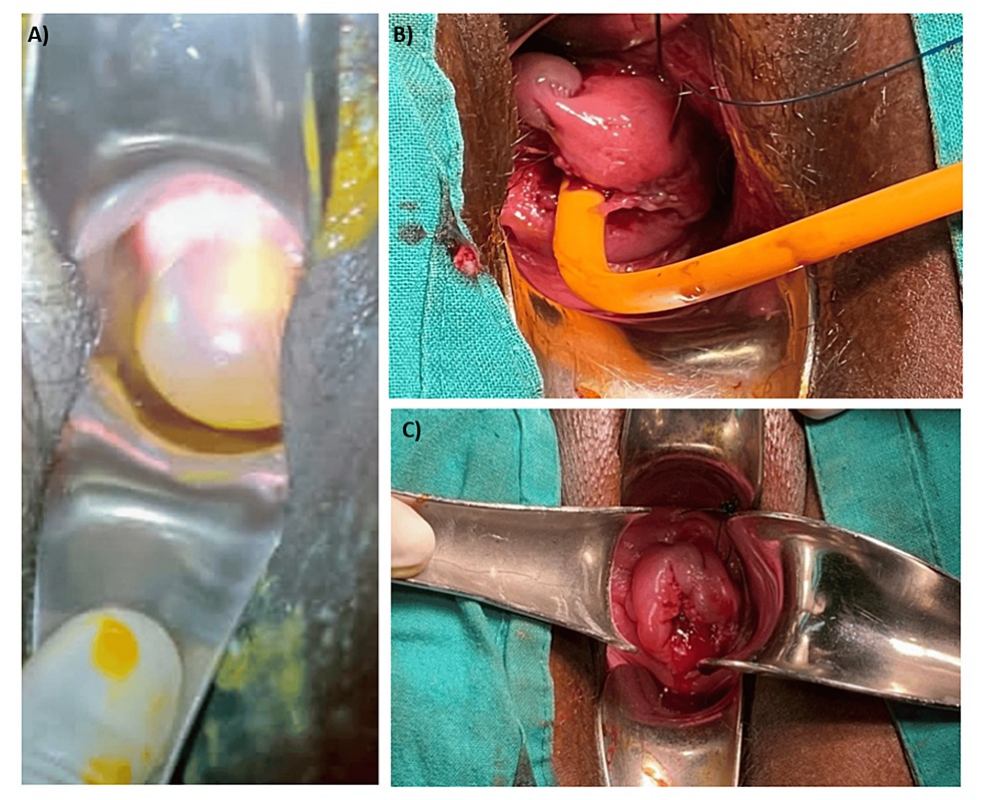
Preoperative, intraoperative, and postoperative images. (A) Preoperative image showing the bulging membrane. (B) Intraoperative image showing a McDonald’s Stitch being taken. (C) Postoperative image showing the McDonald’s stitch in situ.

The patient underwent a cervical cerclage procedure after receiving comprehensive information about the associated risks and benefits, followed by obtaining written consent. The procedure was planned under spinal anesthesia, with the patient positioned in lithotomy. A Foley catheter (size 14) was inserted for urinary catheterization. The cervical lips were gently held using sponge-holding forceps, and careful traction was applied. Amniotic membranes protruding through the cervix were reduced using a gauze covered with sterile rubber gloves soaked in povidone-iodine solution and held with sponge-holding forceps. The bulging membranes were held in position using the inflated balloon of a Foley catheter filled with 30 mL of normal saline (Figure 2, Panel A).

Cervical cerclage was performed with a McDonald’s stitch using prolene no. 1, creating a stitch loop (Figure 2, Panels B, C). The Foley catheter in the cervix was then deflated and removed. In the postoperative period, the patient received intravenous antibiotics and a 40 mg isoxsuprine drip over four to six hours. Following the isoxsuprine drip, the patient was prescribed tablet nifedipine retard 20 mg twice daily and tablet Duphaston 20mg twice daily. Additionally, the patient received injection hydroxyprogesterone caproate 500 mg intramuscularly weekly for three doses. The comprehensive postoperative care and medication regimen aimed to support and maintain the pregnancy after the cerclage procedure.

The patient was discharged after the cervical cerclage procedure and received routine follow-up care until 34 weeks and two days of gestation as per the first-trimester ultrasound when the patient presented with lower back pain and abdominal pain, prompting the removal of the cervical stitch.

However, at 36 weeks and two days, the patient returned with abdominal pain and lower abdominal pain, revealing that the cervix was 3 cm dilated and 60% effaced. Due to a non-reassuring non-stress test, the patient was promptly shifted for an emergency lower-segment cesarean section. The surgical intervention resulted in the successful delivery of a healthy male child weighing 2.5 kg. This comprehensive management approach, including the cervical cerclage and timely intervention, contributed to a positive outcome for both the mother and the newborn.

## Discussion

The predominant etiological factors for cervical insufficiency include inflammation or infection. Acquired causes encompass cervical trauma resulting from events such as vaginal delivery, cervical conization, loop electrosurgical excision procedure, or any forced cervical dilatation, particularly during a prior dilation and evacuation procedure in the context of abortion. Additionally, defective embryological development of Müllerian ducts can be considered a congenital cause [2].

Patients presenting with painless cervical dilatation, indicative of cervical insufficiency, during the second trimester are confronted with two management options: one involves expectant measures, such as bed rest, while the other involves the implementation of rescue cervical cerclage. Despite the limited randomized controlled trials and studies comparing the outcomes between rescue cerclage and expectant management (bed rest), several studies have suggested that rescue cerclage potentially extends pregnancy duration by six to nine weeks, as opposed to fewer than four weeks with bed rest [2]. Overall survival after cervical emergency cerclage is about 74%, with a fetal survival of 88% and neonatal survival of 90% [7].

The International Federation of Gynecology and Obstetrics guidelines recommend the consideration of rescue cerclage before 24 weeks, on an individual case basis, in women presenting with membranes exposed and protruding through the cervical os. However, the risk associated with infective morbidity for both the mother and the baby should be diligently taken into account [8].

A history-based cervical cerclage is recommended for women with a history of three or more preterm deliveries and/or mid-trimester losses. For those with a cervical length of less than 25 mm and a history of one or more spontaneous preterm births and/or mid-trimester losses, an ultrasound-based cerclage is advised. In high-risk women without a history of mid-trimester loss or preterm birth, the use of ultrasound-indicated cerclage in the presence of a short cervix may not demonstrate clear benefits. However, in the case of twin pregnancies, the potential advantages are more apparent at shorter cervical lengths (less than 15 mm). In situations where a cerclage has failed following a history or ultrasound-indicated cerclage, abdominal cerclage is a viable option [9,10].

Various cervical cerclage techniques include Shirodkar’s method, a modified Shirodkar’s method, Wurm/Hefner’s stitch, and Benson and Durfee’s method. However, in our case, the effacement was not advanced and the cervical tissue was favorable, therefore, McDonald’s method was chosen [11].

Various therapeutic approaches have been proposed before or at the time of cervical cerclage. These encompass tocolysis (typically involving indomethacin), administration of antibiotics, and amnioreduction. It is important to note that there is a lack of robust prospective evidence supporting the efficacy of these interventions, and their consideration should be based on the specific circumstances of each case [12].

Current recommendations from the National Institute of Clinical Excellence propose considering the application of rescue cervical cerclage in women exhibiting a dilated cervix and exposed, unruptured fetal membranes, especially between the 16th and 27th weeks of gestation. Nevertheless, it is important to emphasize that the cervical cerclage is more beneficial when implemented during earlier stages of gestation [13].

A lacuna in the current literature is the absence of reports regarding conducting an ultrasound examination between the nuchal translucency scan and the anomaly scan, ideally around 16 weeks. This additional ultrasound may be crucial for detecting cervical incompetence promptly and enabling timely intervention if such a condition is identified.

It is said that pregnancy is a miraculous journey filled with hope, anticipation, and unwavering faith. It was this motherly instinct and profound belief in the potential of the dedicated doctors by the mother to navigate any unforeseen challenges that inspired us to play a pivotal role, thus nurturing the mother’s confidence in our ability to safely deliver her baby. It is aptly said that the bond between an expectant mother and her healthcare team is one of mutual trust and reliance.

## Conclusions

The implementation of rescue cervical cerclage has been shown to be a safe and straightforward surgical intervention capable of extending pregnancy to a viable stage, even in the presence of significant cervical alterations. This procedure is recommended for antenatal women exhibiting advanced cervical changes, following a comprehensive analysis of the overall clinical presentation. In cases of painless cervical dilatation and protrusion of fetal membranes, emergency cerclage effectively reduces the incidence of preterm birth, thereby enhancing gestational age and promoting the survival of newborns. Importantly, the procedure does not elevate the risk of chorioamnionitis or preterm premature rupture of membranes.
